# Supplementary material for: Mechanisms governing the pioneering and redistribution capabilities of the non-classical pioneer PU.1
Source: Nat Commun. 2020 Jan 21;11:402. doi: 10.1038/s41467-019-13960-2 (PMC6972792; doi:10.1038/s41467-019-13960-2)
Supplement: Supplementary file 7 — Source data [file 41467_2019_13960_MOESM7_ESM.zip › Source_Data/FigureS2/FigureS2B_MotifScanOutput/KmeansCluster5/homerResults/motif2.similar.html]

motif2

## Information for motif2

G
C
T
A
C
T
G
A
T
C
A
G
T
A
G
C
T
C
A
G
T
C
A
G
C
T
G
A
G
T
C
A
  
Reverse Opposite:  

C
A
G
T
A
G
C
T
A
G
T
C
A
G
T
C
A
T
C
G
A
G
T
C
G
A
C
T
C
G
A
T
  

|  |  |
| --- | --- |
| p-value: | 1e-527 |
| log p-value: | -1.213e+03 |
| Information Content per bp: | 1.526 |
| Number of Target Sequences with motif | 2539.0 |
| Percentage of Target Sequences with motif | 63.79% |
| Number of Background Sequences with motif | 10632.3 |
| Percentage of Background Sequences with motif | 24.83% |
| Average Position of motif in Targets | 102.2 +/- 46.5bp |
| Average Position of motif in Background | 99.3 +/- 76.0bp |
| Strand Bias (log2 ratio + to - strand density) | -0.0 |
| Multiplicity (# of sites on avg that occur together) | 1.35 |
| Motif File: | file (matrix) reverse opposite |

### Similar de novo motifs found

|  |  |  |  |  |  |  |  |
| --- | --- | --- | --- | --- | --- | --- | --- |
| Rank | Match Score | Redundant Motif | P-value | log P-value | % of Targets | % of Background | Motif file |
| 1 | 0.761 | C A T G C A G T A G T C G T A C G A C T T G A C G C A T C G A T A G C T C A G T | 1e-478 | -1102.876270 | 38.92% | 9.25% | motif file (matrix) |
| 2 | 0.758 | A T C G A C G T A G C T T A G C A C G T A T C G A G T C A C G T A C G T C G A T G T C A | 1e-84 | -194.672614 | 4.17% | 0.41% | motif file (matrix) |
| 3 | 0.751 | C G T A C G T A C G T A A C T G A G T C C G T A A C T G C G T A C G T A | 1e-62 | -144.727170 | 4.50% | 0.78% | motif file (matrix) |
